# Mortality among head trauma patients taking preinjury antithrombotic agents: a retrospective cohort analysis from a Level 1 trauma centre

**DOI:** 10.1186/s12873-016-0094-1

**Published:** 2016-08-02

**Authors:** Sigrid Narum, Odd Brørs, Olav Stokland, Marianne K. Kringen

**Affiliations:** 1 Centre for Psychopharmacology, Diakonhjemmet Hospital, PO Box 85 Vinderen, N-0319 Oslo, Norway; 2 Department of Pharmacology, Oslo University Hospital, Oslo, Norway; 3 Department of Pharmacology, University of Oslo, Oslo, Norway; 4 Former Intensive Care Unit, Oslo University Hospital, Oslo, Norway

**Keywords:** Antithrombotic agents, Warfarin, Platelet inhibitors, Blunt head trauma, Mortality, Older adults

## Abstract

**Background:**

Bleeding represents the most well-known and the most feared complications caused by the use of antithrombotic agents. There is, however, limited documentation whether pre-injury use of antithrombotic agents affects outcome after head trauma. The aim of this study was to define the relationship between the use of preinjury antithrombotic agents and mortality among elderly people sustaining blunt head trauma.

**Methods:**

A retrospective cohort analysis was performed on the hospital based trauma registry at Oslo University Hospital. Patients aged 55 years or older sustaining blunt head trauma between 2004 and 2006 were included. Multivariable logistic regression analyses were used to identify independent predictors of 30-day mortality. Separate analyses were performed for warfarin use and platelet inhibitor use.

**Results:**

Of the 418 patients admitted with a diagnosis of head trauma, 137 (32.8 %) used pre-injury antithrombotic agents (53 warfarin, 80 platelet inhibitors, and 4 both). Seventy patients died (16.7 %); 15 (28.3 %) of the warfarin users, 12 (15.0 %) of the platelet inhibitor users, and two (50 %) with combined use of warfarin and platelet inhibitors, compared to 41 (14.6 %) of the non-users. There was a significant interaction effect between warfarin use and the Triage Revised Trauma Score collected upon the patients’ arrival at the hospital. After adjusting for potential confounders, warfarin use was associated with increased 30-day mortality among patients with normal physiology (adjusted OR 8,3; 95 % CI, 2.0 to 34.8) on admission, but not among patients with physiological derangement on admission. Use of platelet inhibitors was not associated with increased mortality.

**Conclusions:**

The use of warfarin before trauma was associated with increased 30-day mortality among a subset of patients. Use of platelet inhibitors before trauma was not associated with increased mortality. These results indicate that patients on preinjury warfarin may need closer monitoring and follow up after trauma despite normal physiology on admission to the emergency department.

## Background

Bleeding, particularly intracranial bleeding, represents the most well-known and the most feared complications with use of antithrombotic agents, and is associated with a high morbidity and mortality. There is, however, limited documentation whether pre-injury use of antithrombotic agents affects mortality after blunt head trauma. A number of studies have described an increased risk of mortality in head trauma patients on preinjury warfarin [1–5], while other studies report no difference in outcome [6–9]. Studies on outcome after head trauma vary with respect to study design, outcome measurements, and patient characteristics, making comparisons between studies difficult. Most of the previous studies are small (less than 40 individuals using warfarin). Larger studies from administrative- and trauma databases have shown an increased risk of mortality in trauma patients on preinjury warfarin [10–12]. The evidence is conflicting, however, as no increased mortality was seen among subpopulations such as patients 65 years and older with intracranial haemorrhage [10] or among patients with head injury [11]. In some studies, the therapeutic anticoagulation, not the warfarin use itself, has been shown to be important [4, 13–15], indicating that anticoagulants may play a mechanistic role in the adverse outcomes and not serve as markers for co-morbidity that leads to worse outcome.

Whether preinjury use of platelet inhibitors affects mortality after head trauma is even less well studied, and results are conflicting [2, 16–18]. Therefore, further studies exploring the association between preinjury use of antithrombotic agents and trauma outcome are of critical importance.

The objective of this study was to define the relationship between preinjury use of antithrombotic agents and mortality among elderly patients sustaining head trauma.

## Methods

Oslo University Hospital Ulleval (OUH-U) is the major trauma hospital for 550 000 citizens and the trauma referral centre for 2.5 million people. Annually, approximately 1 000 patients of all age groups admitted to OUH-U within 24 h after trauma are registered in the hospital-based trauma registry (Oslo University Hospital Trauma Registry). In this cohort study, patients 55 years or older with blunt head trauma (documented by a diagnosis of concussion, skull laceration or more serious head trauma) were included. Exclusion criteria were penetrating skull injury and intracranial bleeding before trauma. The study inclusion period was 1 January 2004 to 31 December 2006. Death within 30 days after trauma was the primary outcome. Use of antithrombotic agents was the primary exposure of interest. Two separate logistic regression analyses were performed to assess risk factors of mortality among warfarin users and platelet inhibitors users, respectively.

Preinjury use of warfarin, acetylsalicylic acid (ASA), or clopidogrel, as well as the degree of anticoagulation (i.e., International Normalised Ratio, INR) was determined by a review of electronic medical records. No description of antithrombotic agents was considered as non-use. An INR of ≥ 2.0 was considered as therapeutic. Thirty-day mortality was used as main outcome instead of in-hospital mortality because in-hospital mortality can be influenced by early discharge or transfers to other hospitals [19]. The variables assessed as potential confounders on the relationship between antithrombotic drug use and 30-day mortality risk included: age, gender, high- vs low-energy trauma, anatomic injury severity (New Injury Severity Score, NISS), physiological derangement on admission (Triage Revised Trauma Score, T-RTS), and co-morbidity (American Society of Anaesthesiologists Physical Status, ASA-PS). High-energy trauma was defined as motor vehicle and bicycle accidents. Low-energy trauma was defined as falls and other trauma mechanisms. The ASA-PS classification system reflects the co-morbidity existing before the trauma; ASA-PS 1: healthy person, ASA-PS 2: mild systemic disease, ASA-PS 3: severe systemic disease, and ASA-PS 4: severe systemic disease that is a constant threat to life [20]. The variables NISS, T-RTS and ASA-PS have been shown to be independent predictors of survival after trauma [21, 22]. NISS has been shown to be a more accurate method for rating patients with multiple serious injuries in a single body region, e.g., traumatic brain injury, and was thus used instead of the Injury Severity Score (ISS) in the statistical analyses [21, 22]. The T-RTS (range 0–12) is defined as the sum of the clinical category values of respiratory rate (RR), systolic blood pressure (SBT) and Glasgow Coma Scale (GCS), all range 0–4 [22]. Normal physiology was defined as T-RTS = 12, physiological derangement as T-RTS ≤ 11. The T-RTS was used since it has been shown to be a better predictor of outcome than one or more of GCS, RR, and SBP. The variables were collected upon the patients’ arrival at the hospital, but pre-hospital GCS, RR, and SBP scores were used to score T-RTS when in-hospital values were unobtainable.

### Ethics

The Regional Committee for Medical Research Ethics and the Privacy Protection Ombudsman at Oslo University Hospital approved the study. Since data was analysed anonymized, informed consent was waived by the Regional Committee for Medical Research Ethics.

### Statistical analysis

Normally distributed variables were expressed as means ± SD and compared using the student’s *t*-test. The association between patient anticoagulant use and injury characteristics, and outcome (30-day mortality), was assessed using univariate logistic regression with odds ratios (OR) and 95 % confidence intervals (CI) calculated. The analysis was stratified according to warfarin or platelet inhibitor use and checked for confounders and interaction effects. Factors significantly associated with mortality in the univariate analysis were thereby entered into a multivariate stepwise logistic regression. All analyses were performed using the Statistical Package for the Social Sciences (SPSS) for Windows (version 20). For all statistical tests, a *p*-value < 0.05 was considered significant.

## Results

A total of 431 patients aged 55 years or older, admitted to OUH-U within 24 h of trauma during January 2004 to 31 December 2006, were identified. Thirteen patients with penetrating skull injury or with suspected intracranial bleeding before trauma were excluded. This left 418 patients for analysis, 254 (60.8 %) male and 164 (39.2 %) female. The age was normally distributed with mean age 71.4 years. Totally, 156 (37.3 %) patients were defined as having severe preinjury co-morbidity (ASA-PS 3 or 4). In 326 patients (78.0 %), anatomic injury was defined as major trauma (NISS ≥ 15), whereas physiological derangement (T-RTS ≤ 11) was present in 162 patients (38.8 %). Seventy patients (16.7 %) died within 30 days after injury. The 30-day mortality was 23 % (27/116) in 2004, compared to 14 % in 2005 (22/152) and 14 % in 2006 (21/150) (*p* = 0.085). The patients who survived were significantly different from patients who died with respect to age, warfarin use, preinjury morbidity, NISS, ISS, T-RTS, and INR, as shown in Table 1. Falls (257; 61.5 %) and motor vehicle accidents (110; 26.3 %) were the most common trauma mechanisms (Table 2).

**Table 1:** Demographics and clinical characteristics for 418 patients according to outcome

	Dead	Alive	*P* value
Male (%)	39 (15.4)	215 (84.6)	0.343
Female	31 (18.9)	133 (81.1)	
Age mean (SD)	75.8 (10.4)	70.6 (10.4)	0.019
No anticoagulant use (%)	41 (14.6)	240 (85.4)	Default
- Warfarin	15 (28.3)	38 (71.7)	0.014
- Platelet inhibitors	12 (15.0)	68 (85.0)	0.927
- Warfarin and platelet inhibitors	2 (50.0)	2 (50.0)	0.049
High energy trauma	20 (14.4)	119 (85.6)	0.28
Low energy trauma	48 (18.7)	209 (81.3)	
Other mechanisms/unknown	2 (9.1)	20 (90.9)	0.51
ASA-PS mean (SD)^a^	2.4 (1.0)	2.1 (0.9)	0.001
NISS mean (SD)	46.2 (15.7)	25.4 (14.7)	< 0.001
ISS mean (SD)	26.7 (9.5)	18.0 (9.7)	< 0.001
T-RTS mean (SD)	9.5 (2.0)	11.3 (1.3)	< 0.001
INR > 3.5	3 (18.8)	13 (81.3)	< 0.001
INR 2.0–3.5	14 (45.2)	17 (54.8)	
INR < 2.0	20 (20.8)	76 (79.2)	
INR not done	33 (12.0)	242 (88.0)	
ICU days median (IQR)	1.5 (1.0–4.0)	2.0 (1.0–6.0)	0.921

^a^ASA-PS classification was missing for 2 patients

**Table 2:** Sample characteristics by exposure status

	No anticoagulants	Warfarin only	ASA/clopidogrel only	Both warfarin and ASA/clopidogrel
Sample size, *n* (%)	281 (67.2)	53 (12.7)	80 (19.1)	4 (1.0)
Age mean (SD)	69.1 (10.2)	76.3 (9.7)	76.0 (10.0)	80.8 (5.6)
Gender, male (%)	167 (59.4)	39 (73.6)	45 (56.3)	3 (75.0)
Gender, female (%)	114 (40.6)	14 (26.4)	35 (43.8)	1 (25)
ASA-PS (%)^a^				
1	113 (40.2)	4 (7.5)	8 (10)	0
2	105 (37.4)	9 (17.0)	21 (26.3)	0
3 or 4	61 (21.7)	40 (75.5)	51 (63.7)	4 (100)
ISS mean (SD)	19.6 (11.2)	20.6 (5.8)	18.4 (8.6)	22.0 (14.1)
NISS mean (SD)	28.5 (17.2)	30.4 (13.6)	28.3 (15.9)	42.2 (34.3)
RTS median (quartiles)	12.0 (10.0–12.0)	12.0 (10.0–12.0)	12.0 (11.0–12.0)	9.5 (8.25–11.5)
INR ≥ 2.0 (%)	5 (1.8)	39 (74.0)	0	3 (75.0)
INR < 2.0 (%)	60 (21.4)	11 (20.8)	24 (30.0)	1 (25.0)
INR not done (%)	216 (76.9)	3 (5.7)	56 (70.0)	0
30-day mortality (%)	41 (14.6)	15 (28.3)	12 (15.0)	2 (50)
Trauma mechanism (%)				
Motor vehicle accidents	83 (29.5)	7 (13.2)	19 (23.8)	1 (25.0)
Bicycle accidents	23 (8.2)	3 (5.7)	3 (3.8)	0
Falls	159 (56.6)	41 (77.4)	54 (67.5)	3 (75.0)
Other mechanisms	13 (4.6)	1 (1.9)	3 (3.8)	0
Unknown	3 (1.1)	1 (1.9)	1 (1.3)	
Falls (%)				
Fall out of bed	3 (1.9)	2 (4.9)	3 (5.6)	0
Fall from standing	71 (44.7)	28 (68.3)	30 (55.6)	1 (33.3)
Fall in stairs	41 (25.8)	8 (19.5)	11 (20.4)	1 (33.3)
Fall from heights	37 (23.3)	2 (4.9)	8 (14.8)	0
Other falls	7 (4.4)	1 (2.4)	2 (3.7)	1 (33.3)

^a^ASA-PS classification was missing for 2 patients

Of the 137 (32.8 %) patients using antithrombotic agents, 53 used warfarin, 80 used platelet inhibitors (73 ASA, 3 clopidogrel, 4 both), and 4 patients used both warfarin and platelet inhibitors (2 warfarin/ASA, 1 warfarin /clopidogrel, 1 warfarin / both). The corresponding 30-day mortality according to clinical characteristics is shown in Table 1. Table 2 shows demographics and trauma mechanisms according to exposure status. The warfarin and the platelet inhibitor users (mean age 76.6 and 76.0 years, respectively) were significantly older than the non-users (69.1 years) (*p* < 0.001 for both) and had higher preinjury morbidity (mean ASA-PS 2.81 and 2.58, respectively) compared to the non-users (ASA-PS 1.84, both *p* < 0.001). The primary indications for warfarin use were atrial fibrillation in 25 patients, cardiac valve replacement in 13 patients, previous deep vein thrombosis/lung embolism in 6 patients, and myocardial infarction in 2 patients.

Separate logistic regression analyses were performed to evaluate the effect of warfarin and platelet inhibitors on 30-day mortality. The four patients who used both warfarin and platelet inhibitors were excluded from the analyses.

Preinjury warfarin use was a significant predictor of 30-day mortality in the univariate logistic regression analysis, together with older age, anatomic severity (higher NISS), physiological derangement (lower T-RTS) on admission, and pre-injury co-morbidity (higher ASA-PS). Gender and trauma mechanism did not affect the outcome. There was a significant interaction effect between warfarin and T-RTS, suggesting that among patients with physiological derangement (T-RTS ≤ 11) on admission, mortality was to a lesser degree predicted by warfarin (Table 3). In the multivariate logistic model (Table 4), ORs for predictors of 30-day mortality are presented separately for patients with normal physiology (T-RTS = 12) and patients with physiological derangement (T-RTS ≤ 11).

**Table 3:** Warfarin and effect of confounding

	Dead	Dead	Alive	Alive			
	Warf	No warf	Warf	No warf	OR (95 %)	OR MH (95 %)	Breslow-Day Inter-action
Crude	15	41	38	240	2.311 (1.167, 4.576)		
T-RTS						X	0.002
12	8	4	24	161	13.417 (3.751, 47.992)		
11 or less	7	37	13	79	1.150 (0.424, 3.120)		

*Warf* warfarin, *OR* odds ratio, *MH* Mantel Haenzel

**Table 4:** Logistic regression models

	Crude		Adjusted	
	OR (95 % CI)	*P* value	OR (95 % CI)	*P* value
Warfarin with normal physiology (T-RTS = 12)				
Age	1.138 (1.062, 1.219)	< 0.001	1.125 (1.040, 1.216)	0.003
Gender	0.785 (0.228, 2.701)	0.701		
Warfarin	13.417 (3.751, 47.992)	< 0.001	8.278 (1.969, 34.794)	0.004
Trauma mechanism	0.500 (0.131, 1.908)	0.310		
ASA-PS	3.707 (1.630, 8.428)	0.002		
NISS	1.088 (1.028, 1.153)	0.004	1.088 (1.014, 1.168)	0.019
Warfarin with physiological derangement (T-RTS ≤ 11)				
Age	1.066 (1.024, 1.110)	0.002	1.090 (1.039, 1.143)	< 0.001
Gender	1.887 (0.905, 3.934)	0.090		
Warfarin	1.150 (0.424, 3.120)	0.784		
Trauma mechanism	1.065 (0.483, 2.347)	0.877		
ASA-PS	1.257 (0.862, 1.833)	0.234		
NISS	1.056 (1.028, 1.084)	< 0.001	1.064 (1.035, 1.095)	< 0.001
Platelet inhibitors				
Age	1.036 (1.008, 1.064)	0.010	1.081 (1.039, 1.123)	< 0.001
Gender	1.583 (0.882, 2.842)	0.124		
Platelet inhibitors	1.033 (0.514, 2.075)	0.927		
Trauma mechanism	0.838 (0.450, 1.561)	0.578		
ASA-PS	1.304 (0.927, 1.834)	0.128		
NISS	1.089 (1.065, 1.114)	< 0.001	1.070 (1.042, 1.097)	< 0.001
T-RTS	0.483 (0.396, 0.590)	< 0.001	0.582 (0.453, 0.749)	< 0.001

For patients with T-RTS ≤ 11 on admission, the unadjusted mortality was 35.0 % (7/20) and 31.9 % (37/116) among patients on warfarin and patients on no antithrombotic agents, respectively. Among patients with normal physiology on admission, use of warfarin increased the mortality eight times, as 25.0 % (8/32) of patients on warfarin died compared to 2.4 % (4/165) of patients on no antithrombotic agents. Very similar results were found when INR was included in the regression analysis instead of warfarin (results not shown). There was an interaction between therapeutic INR and T-RTS, with 29.0 % mortality among patients with INR ≥ 2.0 and normal physiology compared to only 2.9 % mortality among patients with INR < 2.0.

Preinjury platelet inhibitor use was not a significant predictor of 30-day mortality in the univariate logistic regression analysis. In the multivariate logistic model (Table 4), significant predictors of mortality for preinjury platelet inhibitor users were older age, anatomic severity (higher NISS), and physiological derangement (lower T-RTS).

## Discussion

Outcome after head trauma may be affected by injury mechanisms, patient characteristics, quality of care, and potentially interactions between them. Our study supports previous studies reporting that preinjury use of warfarin, but not platelet inhibitors, are associated with increased mortality following head trauma. In addition, our study indicates that the effect of warfarin on mortality might be dependent on physiological status. Among patients with normal physiology on admission (T-RTS = 12), warfarin use was associated with increased risk of mortality compared to use of no anticoagulants (25 % vs 2 %, respectively). Among patients with physiological derangement (T-RTS ≤ 11) on admission, in contrast, equally high risk of mortality was found among patients with preinjury use of warfarin and those who did not use anticoagulants (35 % vs 32 %, respectively). It thus seems like the negative impact of preinjury warfarin use on mortality is most pronounced in a subpopulation of patients with severe trauma and with normal physiology on admission. Trauma care providers should be aware of this increased mortality risk among head trauma patients on warfarin presenting with normal physiology. Our result is in accordance with a retrospective review including patients with fall from standing [23], and a more recently published study on outcome after head trauma in Medicare beneficiaries [24].

Two recently published large cohort studies, in contrast, showed no increased mortality risk in subpopulations of warfarin users 65 years and older with intracranial haemorrhage [10] or among patients with head injury [11]. Those studies, however, included patients with an expected lower mortality risk, such as younger patients and patients with non-head traumas.

Preinjury morbidity has been shown to be an independent predictor of mortality in the NORMIT model [20, 22]. Surprisingly, co-morbidity (ASA-PS) was not independently associated with mortality in our analysis, and we did not find any interaction effect between NISS and co-morbidity. In our study, more than one third of the patients were classified as having severe systemic disease (ASA-PS 3 or 4), in contrast to 10 % with severe systemic disease found in the study by Jones et al. Differences in study populations may explain the lack of effect of co-morbidity on mortality in our study.

Use of platelet inhibitors was not associated with increased mortality. Studies of antiplatelet use and outcome after head trauma are sparse, the studies are small and results are conflicting [2, 7, 12, 17, 25, 26]. The explanation for this lack of knowledge is complex, but main reasons are lack of information of antiplatelet use in the patient administrative databases, use of over-the-counter platelet inhibitors and use of drugs with platelet inhibition as a pleiotropic effect. The difficulty in assessment of platelet activity through laboratory tests, and the assumption of a smaller bleeding risk compared to warfarin use as seen in other settings, may contribute to the lack of knowledge. This indicates a smaller effect (if any) of platelet inhibition on outcome after head trauma compared to warfarin use.

The strengths of this present study include information regarding the assessment of preinjury morbidity, description of trauma mechanisms, severity of the injury, and the degree of anticoagulation among the majority of the anti-coagulated patients. In studies on trauma outcome, several outcome measures are used such as in-hospital mortality, mortality by end of somatic care, and mortality at 30 days after trauma. We used 30-day mortality since this has been validated in the currently used trauma databank, and has been shown to be the best outcome measure after blunt trauma [19]. Patients intubated before hospital arrival have often been excluded from studies due to lack of T-RTS data. In the OUH-U Trauma Registry the GCS and RR scores recorded before intubation could also be used to include patients intubated before arrival. This means that no group is systematically excluded in this analysis. This study has a retrospective cohort design. Other head-trauma studies, in contrast, have used (matched) case control design [2, 6, 9]. Both study types, with their advantages and disadvantages, may be used to explore the relationship between warfarin use, head trauma and mortality. If warfarin causes more intracranial bleeding, adjustment for injury severity in a matched case control design may cause the differences to disappear because the scoring of anatomic severity accounts for the size of intracranial hematomas.

The main limitation of this study is the retrospective design, which precludes the ability to establish a causal relationship between the risk factors and the outcome. Likewise, there is a possibility of sampling biases if patients on antithrombotic agents or with supra-therapeutic INR are less likely to be transferred from the local hospital to the trauma referral centre due to more severe head injury with prospected poor outcome. A lower mortality among patients with supra-therapeutic INR compared to patients with INR in the therapeutic range may indicate a sampling bias, which would make us underestimate the true mortality risk with antithrombotic agents. An association between the degree of anticoagulation and mortality has been found in some studies of outcome after trauma [4, 13], but not in other studies [7, 8]. This may be explained by a high (almost 50 %) occurrence of sub-therapeutic INR at the time of trauma in these studies [7, 8]. Trauma-induced coagulopathy in patients with severe trauma has been associated with increased mortality, and the incidence of coagulation disorders and subsequent mortality has been particularly high following traumatic brain injury [27]. In our study, we do not know if the non-users of warfarin with therapeutic INR had trauma-induced coagulopathy or were undisclosed warfarin users, or if the warfarin users had trauma-induced coagulopathy in addition to the warfarin induced coagulopathy. Further, we had no access to descriptions of intracranial pathology or cause of death. Such descriptions would have provided deeper insight into trauma severity, however, intracranial pathology is at least partly accounted for in the anatomic trauma score (NISS). Another limitation is the sample size. Despite OUH-U is a trauma referral centre for 2.5 million people, only 418 patients with blunt head trauma of 55 years or older fitted the inclusion criteria during a three year period. The small sample size may thus contribute to uncertain estimates as seen by the wide confidence intervals. This is also a single-centre review whose findings may not be applicable to other centres. In these data, the relationship between use of warfarin and increased mortality persisted after controlling for covariates available in the trauma databank. Whether there are other unmeasured confounders that account for these findings rather than therapeutic anticoagulation, is unknown.

Assessment of preinjury use of warfarin or platelet inhibitors was based on information in the patient records. Thus, underreporting or misclassification may exist. The misclassification is probably larger for the platelet inhibitor use, since there was no assessment of platelet activity. Use of additional drugs affecting platelets, such as nonsteroidal anti-inflammatory drugs, serotonin transporter or receptor inhibitors, or over-the counter ASA, was sparsely reported in the patient records and may explain the lack of effects of platelet inhibitors on mortality.

## Conclusions

In conclusion, our data suggest that use of warfarin is associated with an increased 30-day mortality following blunt head trauma among the subset of patients with normal physiology at admission. Among patients with physiological derangement at admission, about a third of all patients died independently of warfarin use. In contrast, the use of platelet inhibitors is not associated with increased mortality. The result may indicate that patients with severe trauma and preinjury use of warfarin need closer monitoring and follow up after trauma despite normal physiology on admission.

## Abbreviations

ASA, acetylsalicylic acid; ASA-PS, American Society of Anaesthesiologists Physical Status; GCS, Glasgow Coma Scale; INR, International Normalised Ratio; NISS, New Injury Severity Score; NORMIT, Norwegian survival prediction model in trauma; OUH-U, Oslo University Hospital Ulleval; RR, respiratory rate; SBP, systolic blood pressure; T-RTS, Triage Revised Trauma Score
